# Encephalitis: a rare neurological manifestation of mpox-a narrative review

**DOI:** 10.11604/pamj.supp.2025.50.1.46671

**Published:** 2025-06-30

**Authors:** Karpal Singh Sohal, Alvin Acas Miranda, Ashu Michael Agbor

**Affiliations:** 1 Department of Oral Health Services, Muhimbili National Hospital, Dar es Salaam, Tanzania,; 2 Department of Oral and Maxillofacial Surgery, Muhimbili University of Health and Allied Sciences, Dar es Salaam, Tanzania,; 3 School of Dentistry, University of Montagnes, Bangangte, Cameroon

**Keywords:** Mpox encephalitis, neurological manifestations, mpox virus

## Abstract

The Monkeypox virus (MPXV) is a re-emerging global health threat, and since January 2022, it has been reported in 127 Member States across all 6 WHO regions. The recent epidemics have seen a difference in the usual presentation of the disease, ranging from asymptomatic and undetected cases to affecting numerous organ systems. Moreover, MPXV has been shown to cause severe neurological symptoms such as seizures and encephalitis. Therefore, this review article focuses on the evidence-based available literature on mpox infections, highlighting and summarizing encephalitis as a rare neurological manifestation of the disease that may otherwise be overlooked in general practice.

## Introduction

Mpox infection is a zoonotic disease caused by the mpox virus (MPXV), an orthopoxvirus belonging to the Poxviridae family, closely related to the smallpox virus [1,2]. Though the virus was originally isolated in laboratory monkeys in 1958, the first case of human monkeypox virus (HMPX) was confirmed in 1970 in the Democratic Republic of Congo [1-3]. The disease had remained endemic to the African subcontinent until 2003, when it was diagnosed outside the African countries for the first time [1-3]. The MPXV is a re-emerging global health threat since it expanded through borders and has spread over 110 countries, including many non-endemic areas such as Europe and the Americas [2]. This has led to the World Health Organization (WHO) declaring it a public health emergency of international concern in July 2022 [4]. Since January 1, 2022, mpox cases have been reported in 127 Member States across all 6 WHO regions, and as of 30 November 2024, a total of 117,663 laboratory-confirmed cases and 263 deaths had been reported to WHO [5]. The most affected countries accounting for almost 80% of the reported cases globally are: United States of America (n = 34,349), Brazil (n = 13,236), Democratic Republic of the Congo (n = 10,492), Spain (n = 8,443), France (n = 4,371), Colombia (n = 4,280), Mexico (n = 4,192), the United Kingdom (n = 4,146), Germany (n = 4,040), and Peru (n = 3 949) [5].

It is speculated that HMPX spreads primarily from an infected animal to a human by direct contact (touch, bite, scratch, or eating the meat of infected animals) or indirect contact [6]. Human-to-human transmission of the virus may occur through the respiratory system, mucosal membranes, or compromised skin. This mode of transmission includes the inhalation of respiratory droplets, direct contact with skin lesions from infected individuals, as well as contact with contaminated materials, commonly referred to as fomites [6,7]. Other forms of transmission reported include nosocomial transmission, placental (congenital monkeypox), and sexual contact [6].

Mpox’s typical clinical presentation in previous outbreaks was analogous to a slightly different form of variola (smallpox), though with decreased severity and fatality [1,6]. Individuals affected typically initially encounter early signs like fever, headaches, fatigue, and myalgia [1,2,4,8]. A rash typically appears 1-3 days (progressing from macules, papules, vesicles, pustules, to crusts) after symptom onset, primarily on the face and extremities [4,6]. The rash in mpox may resemble varicella (chickenpox); however, lymphadenopathy is an additional feature of mpox that distinguishes it from other similar diseases, such as smallpox, chickenpox, or measles [1,6].

The recent epidemics have seen a difference in the usual presentation (cutaneous manifestation) of the disease, ranging from asymptomatic and undetected cases to affecting numerous organ systems, including the liver, pancreas, and pulmonary systems [1,7]. Moreover, MPXV has the potential to exert common neurological symptoms like headache, and even more severe neurological symptoms such as seizure and encephalitis [2,7]. Therefore, this review article aims to explore the available literature on mpox infections, highlighting and summarizing encephalitis as a rare neurological manifestation of the disease that may otherwise be overlooked in general practice.

## Methods

**Eligibility criteria:** in composing this review, we applied a range of inclusion and exclusion standards. The criteria for inclusion comprised: (a) original research, brief communications, review articles, case reports, and case series focused on the neurological manifestations of mpox; (b) studies that were published in English; and (c) articles released between January 1970 and December 2024.

**Information sources:** we searched the following databases for relevant articles: PubMed, African Journals Online, Scopus, Google Scholar, and ScienceDirect. The final search was carried out on 20th January 2025.

**Search strategy:** the search strategy included simple and combined keywords: “Monkeypox”, “mpox”, “HMPX”, “mpox encephalitis”, “mpox outbreak”, “viral encephalitis”, “mpox virus”, “neurological manifestation”, and “systemic manifestation”.

**Figure 1:**
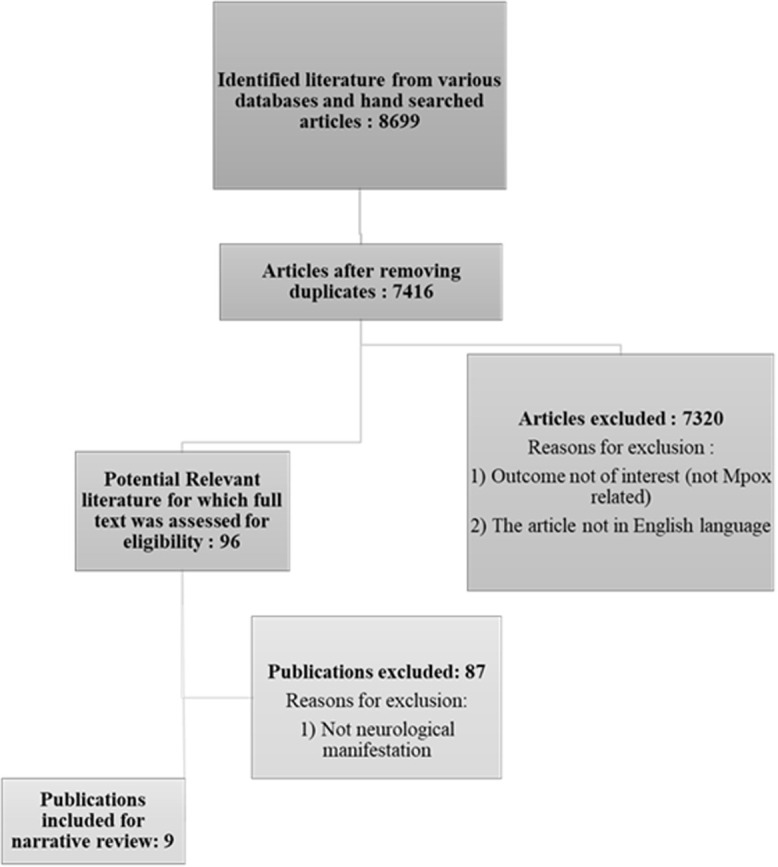
PRISMA flow chart showing search strategies and screening

**Study selection process:** reviewer pairs independently screened titles and abstracts of citations to assess eligibility for inclusion. Full-text publications were then reviewed to finalize inclusion decisions. Discrepancies in eligibility assessments were resolved through discussion and consensus. If a consensus was not reached, a third reviewer arbitrated. During data collection and analysis, the authors worked together by communicating personally, through emails, and on social media platforms (WhatsApp). Ninety-six peer-reviewed articles about mpox were retrieved, and 9 references were included in the final review of neurological manifestations (Figure 1).

**Data item:** the data of interest included the age and sex of the patient, underlying medical condition, duration of hospital stay, and outcome (death or discharge home).

## Results

**General neurological manifestations of mpox:** there are limited studies dealing with the neurological manifestations of HMPX; thus, few neurological complications of the disease have been described in the literature [9]. A recent meta-analysis study by Badenoch et al. [10] pointed out that serious neurological manifestations of MPOX were present in up to 3% of infected individuals. The spectrum of neurological manifestations extends from general prodromal features observed in viral infections like headache, myalgia, and fatigue to severe and rare neurological symptoms such as seizure and encephalitis [2,7]. Headache is the leading symptom with a pooled prevalence of 31%, followed by myalgia and fatigue with a reported pooled prevalence of 27.5% and 17.7% respectively [11]. The potential underlying mechanisms of (mpox) neuropsychiatric manifestations may be a direct central nervous system (CNS) infection, an immune-mediated response, and a psychological reaction to illness [10].

## Discussion

Mpox encephalitis: encephalitis refers to inflammation of the brain parenchyma caused by infection or autoimmunity that results in neurologic dysfunction [12]. When the cause of the inflammation of brain parenchyma is a virus infection, it is termed viral encephalitis (VE) [13]. Viral encephalitis seriously threatens global public health, and its etiology varies according to the geographical region [13,14]. For example, herpes simplex virus was the common cause in Australia, Italy, India, and the United States, whereas in Vietnam, the Japanese encephalitis virus was the most common cause [14]. Central nervous system findings indicative of inflammation are deemed uncommon in mpox infections; however, throughout the present outbreak, a small number of fatal cases, along with very few instances of encephalitis, have been documented, with a mortality rate varying from 1 to 10% depending on the specific clade involved [15].

Probable transmission routes for entry of MPXV and its spread in the brain tissue: though animal studies have shown that MPXV has the potential to cross the blood-brain barrier (BBB), the neurotropic feature of MPXV on human subjects has not been fully understood [16]. The potential route of MPXV to invade the nervous system can be through direct neuro-invasion, the olfactory route, hematogenous spread, and immune-mediated responses [17,18]. Once the virus enters the brain tissue, Schultz-Pernice et al. [19]. Using an advanced in vitro model that closely resembles the human brain, researchers discovered that MPXV primarily spreads from cell to cell by taking advantage of various mechanisms within human neural tissue. Among these mechanisms, viral particles can be transmitted from one cell to another via axons and dendrites.

Diagnosing encephalitis clinically: once the virus invades the nervous system, it triggers a cascade of inflammatory responses that can cause neuronal dysfunction, demyelination, and potential long-term neurological deficits [16,17]. The growing awareness of MPXV´s ability to invade the nervous system and the resulting neurological issues highlights the importance of thorough neurological evaluations and continuous clinical monitoring of patients infected with MPXV [16,17]. In clinical settings, encephalitis (including VE) can be diagnosed based on the proposed standardized case definition for encephalitis in the context of epidemiology and research by the International Encephalitis Consortium [12,20]. To diagnose encephalitis, there are one major and six minor criteria, which are: i) the major criterion: altered mental status persisting for 24 hours or more with no other identified cause (this altered mental status may manifest as a reduced or changed level of consciousness, lethargy, or personality changes). ii) The six minor criteria (two required for possible encephalitis; three or more required for probable or con?rmed encephalitis) are: a) Recorded fever of 38°C or higher occurring within 72 hours before or after the presentation. b) Seizures that are generalized or partial and cannot be completely linked to a pre-existing seizure disorder. c) New onset of focal neurologic findings. d) Cerebrospinal fluid (CSF) leukocyte count ≥5 mm3 e) Anomalies in the brain parenchyma observed on neuroimaging indicating encephalitis that is either a recent development compared to previous studies or seems to have a sudden onset. f) An electroencephalogram shows abnormalities that align with encephalitis and cannot be linked to any other cause. However, Staal and colleagues [20] updated the criteria by adjusting the weight of each criterion, resulting in a sensitivity of 93% for the clinical diagnostic score of possible encephalitis. The sensitivity of probable encephalitis was 51%, maintaining an excellent specificity of 91%.

Documented clinical cases of mpox encephalitis: a recent systematic review by Khan et al. [2] found that the incidence of mpox encephalitis was 0.8%. The literature has few documented clinical cases of mpox encephalitis. The 1st probable case of mpox encephalitis was reported by Jezek et al. [21] during the 1980s outbreak in Zaire (now the Democratic Republic of Congo). Only 1 case in a cluster of 282 patients had mpox encephalitis. The patient was a three-year-old girl who succumbed to the illness the second day after admission to the hospital. A single mpox encephalitis case (out of 34 patients) was reported during the 2003 HMPX outbreak in the United States of America. An otherwise healthy 6-year-old girl suffered from mpox encephalitis and needed to be hospitalized in the intensive care unit and required mechanical ventilation; however, she recovered without significant sequelae after almost 2 weeks of hospitalization [22,23].

In the 2017/18 outbreak of HMPX in Nigeria, out of 40 patients, 3 were diagnosed with mpox encephalitis [24]. Of these 3, two succumbed to their illness. They included a female neonate aged 1 month who died 8 days after diagnosis, and a 43-year-old man who had underlying HIV-1 infection (with CD4 count < 20 cells/μL). During the recent outbreak (2022) of HMPX in the United Kingdom, Cole et al. [25] reported a case of mpox encephalitis in a 35-year-old female patient with no underlying immunodeficiency. The patient was hospitalized for about a month and subsequently recovered from almost all her neurological deficits 3 months after her initial infection.

Sharma et al. [26] reported a case of mpox encephalitis from India in 2023. The patient was a 44-year-old male with a history of high-risk sexual behavior (men having sex with men), on pre-exposure prophylaxis for HIV, and a history of neurosyphilis and genital herpes. He was hospitalized and was diagnosed with mpox encephalitis and neurosyphilis. He was discharged 10 days after admission.Another case from India was reported by Yadav et al. [27] in 2023. The patient was a healthy 22-year-old male who developed signs of HMPX while in the United Arab Emirates, and a few days later, he traveled to India. He was hospitalized after becoming unresponsive at home. Despite the intervention by the medical team, he died 3 days later in the hospital. A case of mpox encephalitis was reported from Sweden by Karin et al. [28] in 2024. Their patient was a 37-year-old man with a history of primary syphilis and was on HIV pre-exposure prophylaxis. The patient had been diagnosed with HMPX about a week earlier. He was subsequently hospitalized and discharged 5 days later following recovery. In a study among HIV-positive patients globally who had been diagnosed with HMPX, Mitja et al. [29] found 1 case of encephalitis. It was in a 44-year-old from the USA with a CD4 count of 106 cells/μL, and on medication. The patient subsequently succumbed to illness 6 weeks after initial symptoms.

Management: currently, there is a minimal understanding of the optimal antiviral treatment for monkeypox disease and its associated complications. The promising options include tecovirimat, cidofovir, brincidofovir, and vaccinia immunoglobulin. Tecovirimat has been preferably used when encephalitis is suspected, as studies in animal models have shown it has the potential to cross the blood-brain barrier [25,26].

## Conclusion

Mpox encephalitis is an atypical and rare but fatal manifestation of the HMPX. The atypical manifestations of mpox stem from a myriad of underlying mechanisms, and this diverse array of factors contributes to the clinical variability [3]. One of the key factors contributing to the pathogenicity of MPXV is its ability to evade the host’s innate immune signaling pathways. This is achieved through the utilization of various immunomodulatory proteins that inhibit the host’s complement system and disrupt the production and signaling of critical antiviral cytokines, including interferons (IFNs) and proinflammatory cytokines [30]. The host’s immunocompromised state can significantly influence the disease’s clinical course and manifestations, leading to atypical presentations, as most affected are immunocompromised, and children [3]. Therefore, doctors should consider mpox encephalitis in cases with inflammation in the presence of atypical vesicopustular rash in high-risk populations in the current outbreak [18]. Diagnosis may involve conducting MPOX PCR, serological tests, immunohistochemistry, and CSF tests apart from the neuroimaging [18,26]. Early initiation of antiviral therapy with tecovirimat is strongly recommended in the early stages of the disease to improve patient outcomes.

### What this study adds

- Mpox is an emerging global infection;
- The rash in mpox may resemble varicella (chickenpox);
- Lymphadenopathy is an additional feature of mpox that distinguishes it from other similar diseases, such as smallpox.

### What is known about this topic

- Mpox encephalitis is a rare neurological manifestation of mpox, with about 10 documented cases to date;
- The length of hospitalization due to mpox encephalitis is long, with mortality of up to 50%;
- Children and immunocompromised adults are at higher risk of getting mpox encephalitis.

## References

[1] Shah J, Saak TM, Desai AN, Gudis DA, Cheema HA, Abuelazm M (2023). Otolaryngologic manifestations among MPOX patients: A systematic review and meta-analysis. Am J Otolaryngol.

[2] Khan SA, Parajuli SB, Rauniyar VK (2023). Neurological manifestations of an emerging zoonosis-Human monkeypox virus: A systematic review. Medicine (Baltimore).

[3] Sokunbi AE, Adeyemi O (2024). Exploring atypical manifestations of Mpox: Anarrative review. Research Journal of Health Sciences.

[4] Gurnani B, Kaur K, Chaudhary S, Balakrishnan H (2023). Ophthalmic manifestations of monkeypox infection. Indian J Ophthalmol.

[5] WHO 2022-24 Mpox (Monkeypox) Outbreak_Global Trends.

[6] Nyame J, Punniyakotti S, Khera K, Pal RS, Varadarajan N, Sharma P (2023). Challenges in the treatment and prevention of monkeypox infection; A comprehensive review. Acta Trop.

[7] Chakravarty N, Hemani D, Paravastu R, Ahmad Z, Palani SN, Arumugaswami V (2024). Mpox Virus and its ocular surface manifestations. Ocular Surface.

[8] Deb N, Roy P, Biswakarma A, Mary T, Mahajan S, Khan J (2023). Neurological Manifestations of Coronavirus Disease 2019 and Monkeypox in Pediatric Patients and Their Management: A State-of-the-Art Systematic Review. Pediatr Neurol.

[9] Billioux BJ, Mbaya OT, Sejvar J, Nath A (2022). Neurologic Complications of Smallpox and Monkeypox: A Review. JAMA Neurol.

[10] Badenoch JB, Conti I, Rengasamy ER, Watson CJ, Butler M, Hussain Z (2022). Neurological and psychiatric presentations associated with human monkeypox virus infection: A systematic review and meta-analysis. EClinicalMedicine.

[11] Satapathy P, Khatib MN, Gaidhane S, Zahiruddin QS, Alrasheed HA, Al-Subaie MF (2024). Multi-organ clinical manifestations of Mpox: an umbrella review of systematic reviews. BMC Infect Dis.

[12] Costa BK, da Sato DK (2020). Viral encephalitis: a practical review on diagnostic approach and treatment. J Pediatr (Rio J).

[13] Li M-L, Chen B-S, Shih S-R (2020). Editorial: Viral Encephalitis. Front Microbiol.

[14] Ai J, Xie Z, Liu G, Chen Z, Yang Y, Li Y (2017). Etiology and prognosis of acute viral encephalitis and meningitis in Chinese children: a multicentre prospective study. BMC Infect Dis.

[15] Marín-Medina DS, Castilla-Gómez L, Poveda M, Ortiz L, Ariza-Serrano LM, Schlesinger-Piedrahita A (2023). Encephalomyelitis in a patient with monkeypox: an unusual complication. J Neurovirol.

[16] Sepehrinezhad A, Ashayeri Ahmadabad R, Sahab-Negah S (2023). Monkeypox virus from neurological complications to neuroinvasive properties: current status and future perspectives. J Neurol.

[17] Thakur A (2024). Re-visiting mpox: Stealth assault on the brain and emerging biomedical research insights. Thakur A. Re-visiting mpox: Stealth Assault on the Brain and Emerging Biomedical Research Insights. Brain Disorders.

[18] Shafaati M, Zandi M (2022). Monkeypox virus neurological manifestations in comparison to other orthopoxviruses. Travel Med Infect Dis.

[19] Schultz-Pernice I, Fahmi A, Brito F, Liniger M, Chiu YC, David T, Oliveira Esteves BI, Golomingi A, Zumkehr B, Gerber M, Jandrasits D (2023). Monkeypox virus spreads from cell-to-cell and leads to neuronal death in human neural organoids. bioRxiv.

[20] Staal SL, Olie SE, van de Beek D, Brouwer MC (2024). Validation of the encephalitis criteria in adults with a suspected central nervous system infection: An updated score. J Infect.

[21] Jezek Z, Szczeniowski M, Paluku KM, Mutombo M (1987). Human Monkeypox: Clinical Features of 282 Patients. J Infect Dis.

[22] Huhn GD, Bauer AM, Yorita K, Graham MB, Sejvar J, Likos A (2005). Clinical Characteristics of Human Monkeypox, and Risk Factors for Severe Disease. Clin Infect Dis.

[23] Sejvar JJ, Chowdary Y, Schomogyi M, Stevens J, Patel J, Karem K (2004). Human Monkeypox Infection: A Family Cluster in the Midwestern United States. J Infect Dis.

[24] Ogoina D, Iroezindu M, James HI, Oladokun R, Yinka-Ogunleye A, Wakama P (2020). Clinical Course and Outcome of Human Monkeypox in Nigeria. Clin Infect Dis.

[25] Cole J, Choudry S, Kular S, Payne T, Akili S, Callaby H (2023). Monkeypox encephalitis with transverse myelitis in a female patient. Lancet Infect Dis.

[26] Sharma R, Nguyen-Luu T, Dhaubhadel P, Sharma A, Naik R (2023). A Rare Co-occurrence of Monkeypox Encephalitis and Neurosyphilis. Cureus.

[27] Yadav PD, Vasu M, Abubaker F, Sahay RR, Reghukumar A, Krishnan AB (2023). An imported case of fatal encephalitis associated with mpox virus infection, India, July 2022. J Med Virol.

[28] Karin H, Ylva B, Sandra S, Aleksandra P, Per B, Klara S (2024). Monkeypox virus-associated meningoencephalitis diagnosed by detection of intrathecal antibody production. BMC Infect Dis.

[29] Mitjà O, Alemany A, Marks M, Mora JI, Rodríguez-Aldama JC, Silva MS (2023). Mpox in people with advanced HIV infection: a global case series. The Lancet.

[30] Hetta HF, Alharbi AA, Alsharif SM, Alkindy TT, Alkhamali A, Albalawi AS (2024). Mpox Virus Infection and Vaccination: Immunopathogenesis and Exploring the Link to Neuropsychiatric Manifestations. Immuno.

